# A Cure for Toothache from Dental Caries

**Published:** 1886-11

**Authors:** 


A Cure for Toothache from Dental Caries.—Dr. V. Gsell-Feltz, of St. Gallen, warmly recommends in toothache from dental caries, the application of cotton-wool soaked in an oily fluid obtained by melting together five parts of camphor, five parts of chloral hydrate and one part of cocaine. Relief is complete and lasting.—*Medical Record.*

---

Dr. T. E. Wright has carefully investigated the question: "When is a man drunk?" and decides as follows: "When consciousness becomes modified, in any degree whatever, through the influence of alcohol, and when, or as long as no exercise of independent nervous force is adequate to restore it to a normal state, the man so affected is drunk."

